# Dietary Intake Assessment of Pre-Packed Graviera Cheese in Greece and Nutritional Characterization Using the Nutri-Score Front of Pack Label Scheme

**DOI:** 10.3390/nu13020295

**Published:** 2021-01-20

**Authors:** Evangelia Katsouri, Emmanuella Magriplis, Antonis Zampelas, Eleftherios H. Drosinos, George-John Nychas

**Affiliations:** 1 Hellenic Food Authority, 11526 Athens, Greece; ekatsouri@efet.gr; 2 Department of Food Science and Human Nutrition, School of Food and Nutritional Sciences, Agricultural University of Athens, 11855 Athens, Greece; emagriplis@aua.gr (E.M.); azampelas@aua.gr (A.Z.); ehd@aua.gr (E.H.D.)

**Keywords:** gravieras, hard cheese, dietary intake, nutrient profile, nutritional labeling, Front of Pack Label (FoPL), nutrient composition

## Abstract

Gravieras are ‘gruyere’ type hard cheeses with a variety of different products and the second highest consumption in Greece. In this study, we present a dietary intake assessment and a nutritional characterization of pre-packed graviera products sold in the Greek market using Nutri-Score Front of Pack Label (FoPL). The nutrient contents of 92 pre-packed graviera products were combined with daily individual consumption data extracted from the Hellenic National Nutrition Health Survey (*n* = 93), attempting to evaluate the contribution of graviera’s consumption to the Greek diet. The analysis of nutrients’ intake as a Reference Intake (RI) percentage ranked saturated fat first on the nutrients’ intake list, with RI percentage ranging from 36.1 to 109.2% for the 95th percentile of consumption. The respective % RI for energy, total fat, carbohydrates, sugars, proteins and salt ranged from 12.7–20.7%, 21.6–50.4%, 0–3.1%, 0–6.1%, 37–57.1% and 6.3–42%. Nutri-Score classified 1% of the products to C—light orange class, 62% to D—orange and 37% to E—dark orange, while no products were classified to A—dark green or B—green classes. The comparison between the Nutri-Score classification and the nutrients’ intake assessment, also separately conducted within the classes, showed a higher salt intake after the consumption of products classified as D—orange and E—dark orange.

## 1. Introduction

According to the Greek National Code of Foodstuffs, Beverages and Objects of Common Use (commonly referred as the “Food Code”), hard and semi-hard cheeses are officially cheese products with a maximum moisture of 30–46% and a minimum fat content of 20–50% on a dry matter basis [1]. Hard and semi-hard cheeses’ category presents a great variety of cheese products with different characteristics, tastes and nutritional values, many of which belong to Greek Protected Designation of Origin (PDO) Products [2], such as specific Gravieras, Kefalograviera, Ladotiri, San Mihali, Kaseri, Batzos, Sfella, and Formaella [3,4]. Among the above cheese products, Graviera is the one with the highest consumption, possessing the second largest market share in the Greek market after feta cheese [5].

Greek graviera is the most abundant hard cheese type category, regarding the variety and the quantity of the products produced and marketed in Greece. Gravieras are hard cheeses with 38% maximum moisture content and 40% minimum fat content on a dry matter basis, manufactured either from sheep’s, goat’s, cow’s or a mixture of these milk types, in various regions in Greece as PDO or non-PDO products. Specifically, most of the gravieras are commercialized with a geographical denomination—under the name of the region where it is produced (graviera of Crete, graviera of Naxos, graviera of Amfilochia, etc.), but only three of them are registered under the Protected Designation of Origin (PDO) EU scheme, including “Graviera Agrafon”, “Graviera Kritis” and “Graviera Naxou” [6,7]. The composition and the sensory properties of the different graviera products may vary substantially depending on the milk type used and the cheese production conditions. Factors such as the animal breed, agroclimatic conditions, season, type of feeding, time of milking, the flora of the local pasture, types of starter cultures used, as well as traditional cheese-making practices comprise sources of product variation [8,9]. Furthermore, many gravieras in Greece are manufactured with the addition of various herbs, spices and other condiments, intentionally used to impart special flavor and color, improve presentation and attractiveness and/or as a source of health-promoting compounds for consumers [10].

Despite the high consumption and market share of graviera cheese in Greece, very limited data are available regarding its nutritional composition and contribution to the individual daily nutrient intake for the Greek population. However, it is well known that dietary intake assessments in nutrition research are crucial in order to correctly reveal the relation between consumption and health, promote consumers’ healthier dietary choices and formulate effective health strategies. Healthy dietary choices have become a priority both for consumers and regulatory authorities. This is mainly due to the fact that the increasing trend of obesity and diet-related non-communicable diseases (NCDs), such as cardiovascular diseases, forms a major cause of premature mortality in Europe. Indeed, in the period 2010–2016, overweight and obesity rates on the continent increased by 2.9% and 2.5%, respectively [11]. Furthermore, NCDs, which are indissolubly related to dietary risk factors, are also leading causes of mortality and disability globally [12,13]. Therefore, curbing the adverse effects of unhealthy diet is a major challenge in developing public health strategies [14].

With regard to the fact that pre-packed foods increasingly comprise the majority of contemporary consumer’s food supplies, food labels’ nutrition declaration, which became mandatory under the Food Information to Consumers (FIC) Regulation [15], constitutes a great tool providing information to consumers and reliable food nutrition data to scientists. In evidence, there is an increasing number of food labelling research studies dealing with nutritional characteristics assessments using food label data [16,17,18]. Regardless of its advantages, however, recent studies have shown that the classic textual information of nutrition labelling has a limited impact on consumers’ dietary choices and is unlikely to lead to any meaningful result from a public policy perspective [19]. In reaction, governments and operators have been experimenting with more effective tools, such as front-of-pack labels (FoP labels or FoPLs) that convey information in a simplified and more salient manner [20]. FoPL has been identified by the Organization for Economic Co-operation and Development (OECD) as the most effective option of food labelling strategy to tackle obesity and provide strong incentives to the agroindustry to reformulate its products in order to improve their nutritional quality [21]. Additionally, the Food and Nutrition Action Plan 2015–2020 of WHO recommends governments to implement FoPLs as part of a policy to address the growing global burden of diet-related NCDs [22]. In accordance with the above potential use of FoPL schemes to help consumers making health-conscious food choices, the European Commission has recently announced that it seems appropriate to introduce a harmonized mandatory FoP nutrition labelling at EU-level, as part of its Farm to Fork Strategy [23]. However, there is still great concern regarding whether an EU-wide nutritional labelling system with a broad food labelling mechanism including nutritional aspects is capable of reflecting the nutritional quality of foods in whole [24]. At the same time, the application of FoPLs in Greek pre-packed foods appears extremely limited and no FoPL has ever been adopted by the Greek Authorities or industry.

Considering all the above, the aim of the present study was to perform an analysis of the nutritional characteristics and dietary intakes of pre-packed graviera cheese in Greece. The objectives of this study were: (a) to comparatively assess the nutritional content of pre-packed graviera products in Greece, (b) to attempt a combination of the nutritional content with consumption data of the Greek population in order to conduct a dietary intake assessment for graviera consumers and evaluate graviera’s contribution to the Greek diet and (c) to evaluate Greek gravieras using Nutri-Score FoPL and discuss its potential use by the Greek Authorities or industry.

## 2. Materials and Methods

### 2.1. Sampling, Labelling Data Collection and Nutritional Content Analysis of Pre-Packed Graviera Products

Sample selection was made after taking into consideration a sufficient geographical representation of the products and their markets, as well as all types of available Greek gravieras’ and brands’ variety. The sample collection of pre-packed cheese products took place in supermarkets, discount and cash and carry chain stores of all major retailers in major Greek cities as well as in online shops, from January 2020 until June 2020.

In total, 92 graviera pre-packed products were identified and collected, 16 of which carried a PDO Geographical Indication mark, including 14 Graviera Kritis PDO and 2 Graviera Naxou PDO products. Regarding non-PDO gravieras (76 products in total), 46 originated from the country’s mainland (Thessaly, Amphilochia, Drama, Macedonia, Peloponnese), 21 from the island of Crete, 5 from the island of Lesvos-Mytilene and 3 from different islands of the Cyclades (Ios, Syros, Paros). Twenty-one of the total 92 products were manufactured with the addition of herbs, spices and other condiments.

All sampled products were purchased and photographed. Data from all the images of all the sides of the package were collected for all products. For each product, all labelling information was retrieved. A photo and labeling information database was created and used for statistical analysis. For each product, all nutrients available on the labeling nutrition declaration table, specifically: energy (kcal/100 g), protein (g), carbohydrates (g), total sugars (g), fat (g), saturated fat (g), and salt (g) per 100 g, were analyzed. Products without a nutritional declaration table were excluded from the analysis.

### 2.2. Statistical Analysis

The data on nutrient contents of graviera products and daily individual consumption extracted from the Hellenic National Nutrition and Health Survey were analyzed using the descriptive statistics option of Microsoft Excel 2003 (Microsoft Corp. Redmond, WA, USA). The 5th, 50th and 95th percentiles were calculated and used to assess the nutrient intakes, which were presented as cumulative distributions or boxplots graphs.

### 2.3. Nutrients Intake Assessment by Graviera Consumption

Individual daily nutrient intakes of healthy adult graviera consumers in Greece were calculated by combining the nutrient contents of the sampled products with graviera cheese consumption data obtained from the Hellenic National Nutrition and Health Survey (HNNHS) database [25]. According to the HNNHS database, 93 adults (43% males) had reported graviera cheese consumption in at least one of the two 24 h recalls conducted. Details on 24 h recall methods have been previously described [25]. The data of graviera’s daily individual consumption were combined with the data of the basic nutrient concentrations of the pre-packed graviera cheese products so as to provide an overall assessment of graviera cheese contribution to the intake of nutrients. As a way to portray variability, the intake of nutrients by the consumption of graviera cheese was calculated using the 5th, 50th and 95th percentiles of both the individual daily consumption and the nutrient content of the 92 tested products. To demonstrate the contribution of graviera cheese consumption to an adult’s diet, the intake of nutrients was also expressed as a percentage of the European Daily Reference Intake (RI) values as set by the European Regulation (EU) 1169/2011 on the provision of food information to consumers [15]. The RI values used were: 2000 kcal, 70 g, 20 g, 260 g, 90 g, 50 g and 6 g for energy, total fat, saturated fat, carbohydrates, sugars, proteins and salt, respectively.

### 2.4. Evaluation of the Nutritional Content of Graviera Products Using the Nutri-Score FoP Label Scheme

The 92 pre-packed graviera products were classified based on their nutritional profile using the Nutri-Score FoP label scheme [26]. A detailed description of the selected FoPL system and its graphical format is presented in Table 1.

Nutri-Score is a color-coded label that provides a summary interpretive indication of the nutritional quality of the food. Based on the content of the product per 100 g, its underlying nutrient profiling system includes both unfavorable-negative nutrients (energy, saturated fat, sugars, and sodium) and favorable-positive elements (fiber, protein, and percentage of fruit, vegetables, legumes, nuts, rapeseed, walnut and olive oil) to yield a summary score (ranging between −15 and 40). The score is finally calculated as the difference (N−P) between negative total (N) and positive total (P) points, and represented in a five-class color-coded scale (with each class expressed by a color and a letter). Products with higher nutritional quality are rated as A (dark green), and products with lower nutritional quality are rated as E (dark orange). The underlying algorithm for Nutri-Score was adapted from the 2005 Food Standards Agency nutrient profiling system [27]. Regarding calcium content, according to Nutri-Score’s modified criteria for cheeses, the protein content is counted. This ensures that their relative calcium content is accounted for, although calcium is not one of the nutrients subject to mandatory declaration [28].

The classification of pre-packed graviera cheese products against Nutri-Score was based on their nutrient contents recorded from labels’ nutrition declaration tables. Nutri-Score estimations were made using the model’s calculation criteria and supportively confirmed randomly through the Open Food Facts project database, which is an international collaborative web project based on a wiki-like interface gathering food composition data based on the available back-of-pack labelling of products [29]. As suggested by other studies [30], the ability of the FoPL to discriminate the nutritional quality of foods is based on the number of available color classes within a group of foods. The more color classes available among the products of a food group-subgroup, the better the discriminating ability of Nutri-Score FoPL was considered.

## 3. Results

### 3.1. Analysis of Nutritional Content of Graviera Products

**Figure 1:**
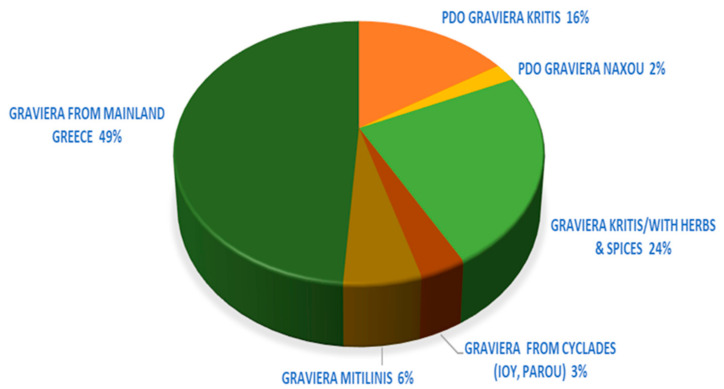
Pie-chart of the origin of all pre-packed gravieras’ with or without a Protected Designation of Origin (PDO) mark, as a percentage of the sum of the products tested in the Greek market.

In total, 92 products of pre-packed graviera cheese were identified in the major Greek retail chains and online shops. According to their labeling information, all products were produced in approved dairy production establishments [31], mainly in five wide regions throughout the country (West Greece and other mainland districts (49%) Crete (40%), North Aegean Islands and basically Lesvos-Mytilene (6%), and South Aegean Islands and basically Cyclades (5%)). Regarding PDO gravieras, Kritis PDO dominates the pre-packed gravieras market with a 16% percentage of abundance, Naxou PDO follows with 2%, while no pre-packed graviera Agrafon PDO products were found in the Greek market (Figure 1).

From the total 92 pre-packed graviera products identified in the market, 83 had a full nutrition declaration on their labels. Two products had an incomplete nutrition declaration and seven products had no nutrition declaration on their labels. Table 2 presents the descriptive statistics of the nutritional content (energy, protein, carbohydrates, total sugars, fat, saturated fat, and salt per 100 g) of the products according to their nutrition declaration on the label.

Overall, the results of the survey showed that the nutritional contents of pre-packed graviera cheese products vary significantly. Specifically, the estimated ranges per 100 g were—energy: 302–492 kcal, total fat: 18–42 g, saturated fat: 8.6–26.0 g, carbohydrates: 0–9.5 g, sugars: 0–6.5 g, proteins: 22–34 g and salt: 0.5–3.0 g. The coefficient of variation (%CV = (Standard Deviation/Mean) ∗ 100) for the different nutrients ranged from almost 8% for energy and protein to 185.4% for sugars. Calcium content ranged between 371 and 910 mg/per 100 g with a median of 600 mg/100 g. It needs to be noted, however, that due to the fact that calcium is not subject to mandatory declaration, only 8 out of 92 products with nutritional tables declared its content in their labelling.

### 3.2. Nutrients’ Intake Assessment by Pre-Packed Graviera Consumption and Comparison with the Respective European RIs

Graviera cheese consumption data for 93 healthy adult Greek consumers, from the HNNHS database [25], were extracted and analyzed. The descriptive statistics of the consumption are presented in Table 3.

**Figure 2:**
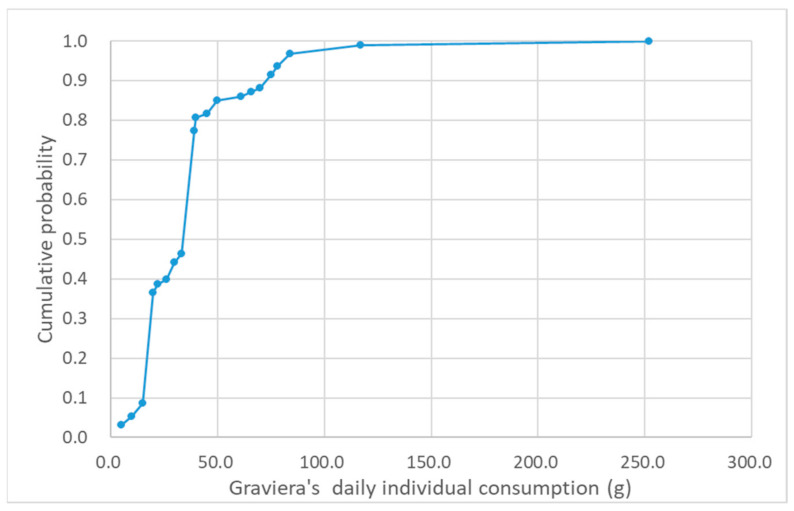
Cumulative frequency graph of Greek adults’ daily individual consumption of graviera (g) based on data of 93 healthy adult Greek consumers extracted from the Hellenic National Nutrition and Health Survey (HNNHS) database.

The cumulative frequency chart of Greek adults consuming graviera cheese (g) per capita and per day is presented in Figure 2.

The results from consumption analysis showed that the consumption of gravieras presents a significant variation—an average value of 38.9 g and a median value of 39.0 g, while consumption per capita and per day ranged from 5 g to 252 g. The estimated % CV was 80.6% and the 5th percentile and 95th percentile were 13 g and 84 g, respectively.

**Figure 3:**
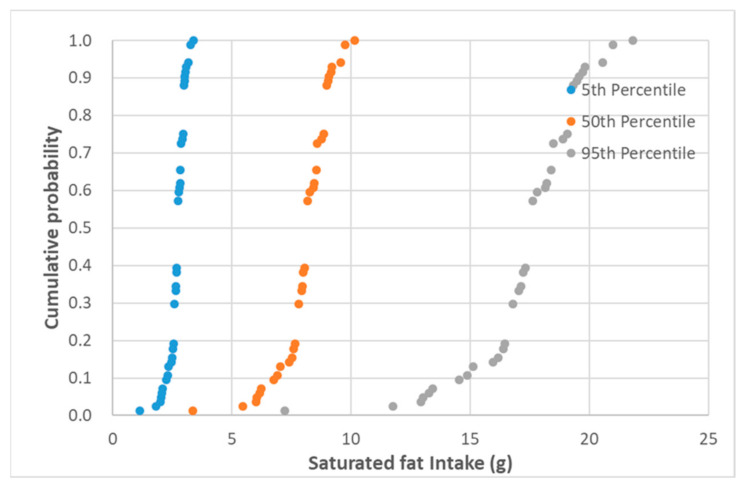
Cumulative probability of saturated fat (g) intake per capita and per day of Greek adults consuming graviera cheese marketed in the Greek market for the 5th, 50th and 95th percentiles of daily consumption.

**Figure 4:**
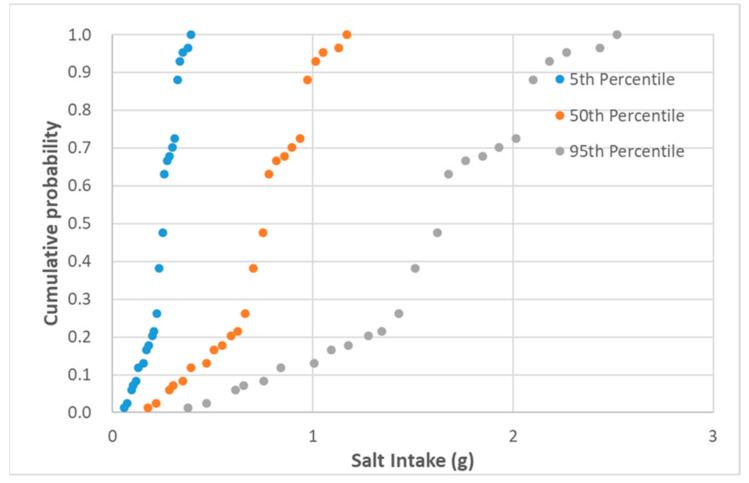
Cumulative probability of salt (g) intake per capita and per day of Greek adults consuming graviera cheese marketed in the Greek market for the 5th, 50th and 95th percentiles of daily consumption.

The data of graviera’s daily individual consumption were combined with the data of the basic nutrient concentrations of the pre-packed graviera cheese products. In the dietary intake assessment, as a part of a nutrition risk analysis, taking into account variability of intake is of great importance [32]. Thus, with a view to assess the variability of both daily consumption and nutrient content among the various products in the present study, the 5th, 50th and 95th percentiles were used. The output of the assessment gives a detailed overview of the variability in the nutrient intake of pre-packed graviera cheese consumers in Greece, which derives from the differences in nutritional content among products sold in the market and the daily consumption quantity among consumers. Denotative cumulative probability graphs of the saturated fat and salt intake per capita and per day of Greek adults consuming graviera cheese marketed in the Greek Market for the 5th, 50th and 95th percentiles of daily consumption are presented in Figure 3 and Figure 4.

**Figure 5:**
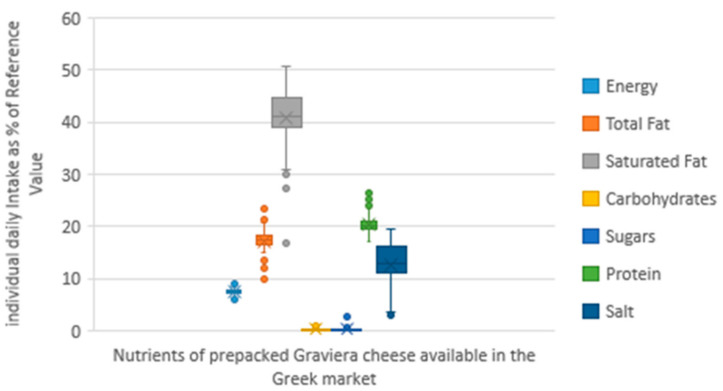
Daily intake per capita as a percentage of European Daily Reference Intakes (RIs) for the 50th percentile of the daily consumption of pre-packed graviera cheese marketed in the Greek market.

**Figure 6:**
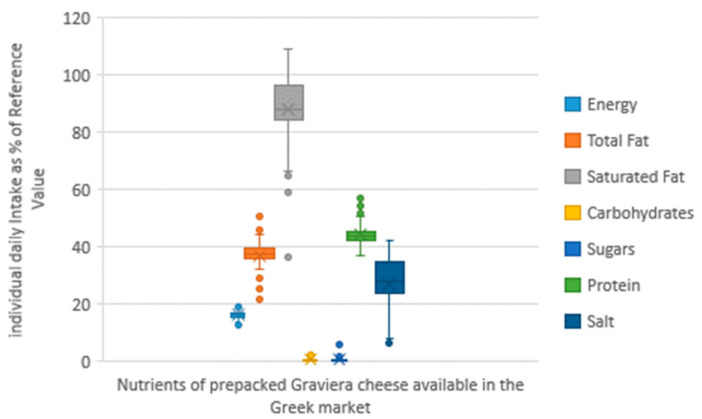
Daily intake per capita as a percentage of European Daily Reference Intakes (RIs) for the 95th percentile of the daily consumption of pre-packed graviera cheese marketed in the Greek market.

The intake of nutrients expressed as a percentage of the European Daily Reference Intake (RI) values is shown in Figure 5 and Figure 6, presenting the boxplots of the daily nutrient intake as an RI percentage by graviera cheese consumption for the 50th and 95th percentiles of daily consumption quantity.

For the 50th percentile of daily individual graviera consumption (corresponding to 39 g), the estimated ranges for energy, total fat, saturated fat, carbohydrates, sugars, proteins and salt were 5.9–9.6%, 10.0–23.4%, 16.8–50.7%, 0.0–1.4%, 0.0–2.8%, 17.2–26.5% and 2.9–19.5%, respectively. For the 95th percentile of daily individual graviera consumption (corresponding to 84 g), the %RI for energy, total fat, saturated fat, carbohydrates, sugars, proteins and salt were 12.7–20.7%, 21.6–50.4%, 36.1–109.2%, 0.0–3.1%, 0.0–6.1%, 37.0–57.1% and 6.3–42.0%, respectively.

### 3.3. Nutrient Profile Evaluation Using Nutri-Score FoP Label Scheme

The 92 pre-packed graviera cheese products were evaluated against the Nutri-Score FoP label scheme. The distribution of graviera cheese products in the different Nutri-Score classes is shown in Table 4.

The results showed that 62% were classified in the D—orange class, 37% of the products were classified as E—dark orange, while only one product (1%) was classified as C—light orange, according to the Nutri-Score classification scale. None of the products were classified as A—dark green or B—green. Overall, three color classes of the Nutri-Score FoPL were found to be available among the products of graviera’s group-subgroup of cheeses.

In order to evaluate the relation between the Nutri-Score output and the nutrients’ intake, the daily intakes of graviera’s nutrients were estimated separately for each group of products classified in the different Nutri-Score classes, for the 5th, 50th and 95th percentiles of daily consumption (Table 5).

**Figure 7:**
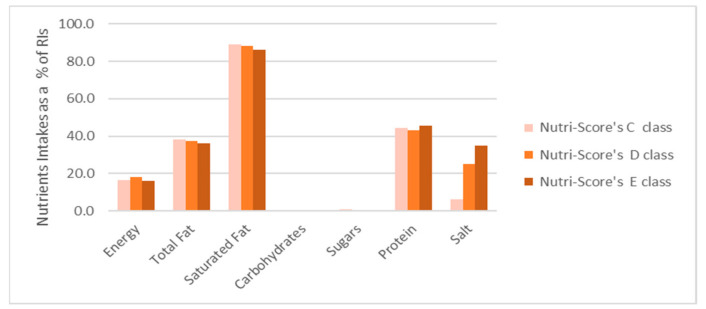
Daily individual nutrient intake as a percentage of European Daily Reference Intakes (RIs) for the 95th percentile of daily consumption of pre-packed graviera cheeses classified by Nutri-Score as C, D and E.

The above assessment showed significant differences in the salt intake among the Nutri-Score classes. For example, in the 95th percentile of daily consumption, the salt intake was 0.4 g, 1.5 g and 2.1 g for cheeses classified as C, D and E, respectively. In contrast to salt, the differences in the daily intake of the rest of the nutrients were small among the Nutri-Score classes. The above conclusions can be seen more clearly in Figure 7, where the daily intakes for each Nutri-Score class are presented as percentages of the European Daily Reference Intake (RI) values as set by the European Regulation (EU) 1169/2011 for the 95th percentile of daily consumption.

Indeed, as shown in the latter figure, while the intake of energy, fat, saturated fat, carbohydrates and protein does not present significant differences among the Nutri-Score classes, the salt intake increases from 6.3% of RI for class C to 35% of RI for class E.

## 4. Discussions

The nutrient content analysis of Greek pre-packed graviera cheese products carried out in the first part of the present study showed a high variability in the nutrient concentrations among products available on the market. This can be ascribed to the differences in the raw material (milk), the predominant microflora of the dairy plants and the cheese-making practices [7]. Despite the above variability, however, average values of nutrient concentrations recorded in the present study were in agreement with previously reported nutrient contents of hard cheeses [33].

In the second part of the study, the nutritional content of graviera cheese was combined with consumption data so as to evaluate the contribution of graviera to the Greek diet. The results showed that the estimated daily intakes of basic nutrients from graviera consumption by a healthy adult can vary significantly, conditional on the consumption quantity and the nutrient content of the consumed product. Comparing the results of the different nutrients, the ranking of daily intakes from pre-packed graviera cheese consumption estimated as a percentage of European RI was (from higher to lower intake): 1—saturated fat, 2—protein, 3—total fat, 4—salt, 5—energy, 6—sugars, 7—carbohydrates. Among them, the highest intake was observed for saturated fat, which may exceed the RI, with percentages up to 109.2% of the RI. The latter indicated that graviera cheese is an important contributor to the saturated fat intake in the Greek diet. This information, better explained in the next paragraph, is important in terms of nutrients’ intake assessment and stays in line with the initial aims of this study.

Graviera and feta are the most highly consumed cheeses in Greece. A comparison of the nutrient intakes from the consumption of the two cheeses shows significant differences. In a previous study, Katsouri et al. [17] reported that for the 95th percentile of daily feta consumption, the %RI for energy, total fat, saturated fat, carbohydrates, sugars, proteins and salt were 11.0–17.2%, 28.5–41.4%, 64.0–101.5%, 0.0–1.2%, 0.0–3.3%, 26.2–42.0% and 20–85%, respectively. Although saturated fat presents the highest intake for both cheeses, graviera consumption results in much lower salt intake and higher protein intake compared to feta cheese. The above comparison indicates that health-associated events related to dairy consumption may differ among product types [34] and stresses the need for nutrient intake analysis of foods as the basis for the development of strategies for nutrition and health. More studies like the present one for a wide range of food products would lead to the development of a complete nutritional database and support the identification and effective selection of strategies and interventions for improved health. Such strategies and interventions may include food reformulation, possible revision of national dietary guidelines, marketing restrictions, industry interventions, the improvement of food label information, and educational campaigns, and some are already in place in several countries of the EU or at EU-level [35].

In the last part of this study, the pre-packed graviera products sold in the Greek market were classified using the Nutri-Score Front of Pack Label (FoPL) scheme. The selection of Nutri-Score FoPL was based on previous studies reporting a very good performance of the scheme regarding increasing consumers’ awareness of food’s nutritional quality, the perception of FoPL and encouraging healthier choices, in different countries and for various food products [30,36,37,38]. More in detail, Nutri-Score was found to perform best compared to other FoPLs—specifically the Health Star Rating system (HSR), Multiple Traffic Lights (MTL), Reference Intakes (RIs), SENS (supported by retailers) and Warning Symbol—as shown in one of the scarce comparative experimental studies [30,39]. Additionally, Nutri-Score has already been adopted in several European countries (France, Belgium, Luxembourg, Netherlands, Denmark, Spain, Germany) as an appropriate tool to facilitate consumers’ understanding of food’s nutritional quality and advance healthier food choices, while several review articles have concluded that FoPLs, in general, are favorably perceived by consumers and can increase their awareness about the healthiness of various food products [30,38]. The results of the present study confirmed the ability of Nutri-Score’s FoPL to scan nutritional variability within a food category and identify nutritional quality [30,37]. The majority of graviera cheese products were classified to the D—orange and E—dark orange classes. The latter classification can be credited to the relatively high levels of saturated fat and salt in graviera cheese, which are evaluated as “negative” in Nutri-Score as well as in all other nutrient profile models due to their association with NCDs. Only one product was classified to the C—light orange class, mainly due to its low salt and high protein concentration (a “positive” factor in Nutri-Score), indicating that this product represents a healthier choice among other graviera cheeses. The analysis of the daily intakes of graviera’s nutrients for each group of products classified in the different Nutri-Score classes confirmed the classification of Nutri-Score, especially in relation to the salt content. Indeed, salt was identified as the most important factor determining the Nutri-Score classification of graviera cheese.

Apart from the advantages of Nutri-Score, though, the above results also impose some skepticism on a potential univocal characterization of the health status of cheeses by an FoPL. Based on the classification performed in the present study, traditional PDO dairy products, such as graviera cheese, which are important components of the European diet and a valuable source of nutrients for humans [40], are classified by the Nutri-Score as “less healthy”. The latter is not consistent with the Greek food-based Dietary Guidelines [41], which suggest that “dairy products are basic food, encouraged to be consumed in up to 2 portions daily, preferably”. Moreover, several studies report a null or inverse relationship between cardiovascular disease risk and mortality and dairy consumption, although there is no clear dose response relationship [42]. These concerns stress the need for further research in order to improve the applicability of nutritional tools such as the Nutri-Score. For example, the inclusion of the daily consumption–portion size and/or the content of other nutrients, such as vitamins D and B12 (for cheeses), could improve the ability of Nutri-Score to characterize the health status of dairy products, including PDO cheeses.

In conclusion, this study follows the concept of dietary exposure assessment as a part of a scientific risk assessment process to support decision-making in the development of nutritional and health mitigation strategies [32]. In the nutritional field, it is generally accepted that food is recognized as having both beneficial and adverse effects on health. Nutrition declaration tables are definitely considered to be an important tool for the presentation and evaluation of food’s nutritional value. However, other complementary schemes and methodologies, such as nutritional FoPL, Nutrient Profile Models and schemes, nutrients’ intake assessments, the nutrient density concept [43] and even the concept of integrated risk-benefit assessments [44], should be further applied in conjunction with regulatory guidance [45] to ensure the promotion of genuinely healthier choices for consumers [46].

## Figures and Tables

**Table 1:** Presentation of the Nutri-Score Front of Pack (FoP) label scheme parameters.

Nutri-Score Parameters	
Categories	solid foods/beverages
Sub-Categories	cheeses/fats, oils
Type	summary-interpretative-colour coded-5 classes scaled from A to E (from healthy to unhealthy)
Calculation Approach	scoring algorithm
Reference Quantity	100 g/100 mL
Unfavorable Elements	energy, saturated fat, sugars, sodium
Favorable Elements	fiber, protein, fruit, vegetables, legumes, nuts, rapeseed oil, walnut oil, olive oil
Purpose of Current Use	FoPL (non mandatory)
Developer	Public
Countries AdoptedNutri-Score	FR, BE, GE, ES, DE, NL, LU
Logo	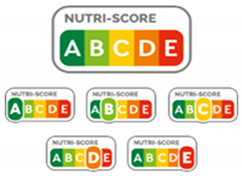

FR: France, BE: Belgium, GE: Germany, ES: Spain, DE: Denmark, NL: Netherlands, LU: Luxembourg.

**Table 2:** Descriptive Statistics of nutrients’ concentrations (per 100 g) of pre-packed graviera cheese products in the Greek market.

	Energy (kJ)	Energy (kcal)	Fat(g)	Saturated Fat (g)	Carbohydrates (g)	Sugars (g)	Protein (g)	Salt (g)	Calcium (mg)
Mean	1620.7	389.4	30.8	20.9	1.2	0.5	26.2	1.9	648.9
Standard Error	13.9	3.4	0.4	0.3	0.2	0.1	0.2	0.1	63.5
Median	1610.0	388.0	31.0	21.0	0.6	0.2	25.9	2.0	600.0
Mode	1537.0	370.0	30.0	21.0	0.1	0.1	25.0	2.0	600.0
Standard Deviation	126.8	31.3	3.6	2.6	1.6	0.9	2.2	0.6	179.5
Kurtosis	2.1	2.0	3.2	5.1	10.1	31.3	2.1	0.0	0.1
Skewness	0.5	0.5	−0.2	−1.5	2.8	4.9	1.2	−0.6	0.4
Range	778.0	190.0	24.0	17.4	9.5	6.5	12.0	2.6	539.0
Minimum	1259.0	302.0	18.0	8.6	0.0	0.0	22.0	0.5	371.0
Maximum	2037.0	492.0	42.0	26.0	9.5	6.5	34.0	3.0	910.0
% CV	7.8	8.0	11.8	12.7	131.0	185.4	8.5	30.6	27.7
Count	83	85	85	84	84	83	84	84	8

%CV= (Standard Deviation/Mean) ∗ 100.

**Table 3:** Descriptive Statistics of graviera cheese consumption data for adults 20–65 years old according to the Hellenic National Nutrition and Health Survey (HNNHS).

Graviera Daily Consumption (g)	
Mean	38.9
Standard Error	3.3
Median	39.0
Mode	39.0
Percentile 5	13.0
Percentile 50	39.0
Percentile 95	84.0
Standard Deviation	31.4
Sample Variance	983.9
Kurtosis	22.9
Asymmetry	3.9
Range	247.0
Minimum	5.0
Maximum	252.0
%CV	80.6
Count	93.0

**Table 4:** Distribution of graviera cheese products in the different Nutri-Score classes.

Nutri-Score FoP Classes	Nutri-Score FoP CriteriaPoints for Solid Food	Average Scores in Products Tested	Range of Scores in Products Tested	Classification According to Estimated Scores, and Percentage of Products in Each Nutri-Score FoP Class
A—dark green	−15 to −1			0%
B—green	0 to 2			0%
C—light orange	3 to10	10	10	1%
D—orange	11 to 18	16	12–18	62%
E—dark orange	19 to 40	19	19–21	37%

**Table 5:** Daily individual intakes of nutrients from the consumption of graviera cheese products classified in different Nutri-Score classes. Intakes are estimated based on the median values of nutrient contents for each class.

Nutrient	Nutri-Score Class								
	C			D			E		
	Consumption Percentile								
	5th	50th	95th	5th	50th	95th	5th	50th	95th
	Daily Nutrient Intake (kcal or g)								
Energy (kcal)	51.5	154.4	332.6	50.6	151.9	327.2	50.1	150.2	323.4
Total Fat (g)	4.1	12.4	26.8	4.0	12.1	26.0	3.9	11.7	25.2
Saturated Fat (g)	2.8	8.3	17.8	2.7	8.2	17.6	2.7	8.0	17.2
Carbohydrate (g)	0.1	0.3	0.6	0.1	0.3	0.6	0.1	0.2	0.4
Sugars (g)	0.1	0.3	0.6	0.0	0.1	0.1	0.0	0.1	0.1
Protein (g)	3.4	10.3	22.1	3.3	10.0	21.6	3.5	10.5	22.7
Salt (g)	0.1	0.2	0.4	0.2	0.7	1.5	0.3	1.0	2.1

## Data Availability

Data used and presented in this study are openly available in the products labels.
